# A Web-Based Training Program Using Cognitive Behavioral Therapy to Alleviate Psychological Distress Among Employees: Randomized Controlled Pilot Trial

**DOI:** 10.2196/resprot.3629

**Published:** 2014-12-02

**Authors:** Makiko Mori, Miyuki Tajima, Risa Kimura, Norio Sasaki, Hironori Somemura, Yukio Ito, June Okanoya, Megumi Yamamoto, Saki Nakamura, Katsutoshi Tanaka

**Affiliations:** ^1^Department of Occupational Mental HealthGraduate School of Medical SciencesKitasato UniversitySagamihara-City KanagawaJapan; ^2^National Center for Cognitive Behavior Therapy and ResearchNational Center of Neurology and PsychiatryKodaira City TokyoJapan

**Keywords:** Web-based training program, cognitive behavioral therapy, stress management, workplace, Internet, group

## Abstract

**Background:**

A number of psychoeducational programs based on cognitive behavioral therapy (CBT) to alleviate psychological distress have been developed for implementation in clinical settings. However, while these programs are considered critical components of stress management education in a workplace setting, they are required to be brief and simple to implement, which can hinder development.

**Objective:**

The intent of the study was to examine the effects of a brief training program based on CBT in alleviating psychological distress among employees and facilitating self-evaluation of stress management skills, including improving the ability to recognize dysfunctional thinking patterns, transform dysfunctional thoughts to functional ones, cope with stress, and solve problems.

**Methods:**

Of the 187 employees at an information technology company in Tokyo, Japan, 168 consented to participate in our non-blinded randomized controlled study. The training group received CBT group education by a qualified CBT expert and 1 month of follow-up Web-based CBT homework. The effects of this educational program on the psychological distress and stress management skills of employees were examined immediately after completion of training and then again after 6 months.

**Results:**

Although the training group did exhibit lower mean scores on the Kessler-6 (K6) scale for psychological distress after 6 months, the difference from the control group was not significant. However, the ability of training group participants to recognize dysfunctional thinking was significantly improved both immediately after training completion and after 6 months. While the ability of participants to cope with stress was not significantly improved immediately after training, improvement was noted after 6 months in the training group. No notable improvements were observed in the ability of participants to transform thoughts from dysfunctional to functional or in problem-solving skills. A sub-analysis of participants who initially exhibited clinically significant psychological distress (K6 score ≥5) showed that the mean K6 score was significantly improved immediately after training completion for the training group compared to the control group (−2.50 vs −0.07; mean difference 2.43, 95% CI 0.55-4.31; *d*=0.61), with this effect remaining even after 6 months (−3.49 vs −0.50; mean difference 2.99, 95% CI 0.70-5.29; *d*=0.60).

**Conclusions:**

Our results suggest that a brief stress management program that combines group CBT education with Web-based CBT homework moderately alleviates the distress of employees with clinically significant psychological distress. In addition, the program might help improve employees’ ability to evaluate their own stress management skills.

## Introduction

Alleviating psychological distress in employees is essential in terms of health and work performance. Epidemiological studies have reported that the proportion of workers whose stress level is high enough to necessitate treatment is approximately 15% [1,2]. Despite this, many experiencing such high levels of stress continue to work without receiving proper care [1]. According to a Japanese national survey, 58% of workers report feeling significantly strong psychological distress [3].

Systematic reviews have reported that work-related psychological distress, regardless of clinical significance, has been found to be related to a number of mental disorders, including depression and anxiety disorders [4,5]. Further, to improve work performance, increasing emphasis is being placed on managing psychological distress regardless of clinical significance [6,7].

In a national survey, 60% of workers reported strong psychological distress, which suggests that other workers might also feel some degree of psychological distress, the Japanese Ministry of Health, Labor and Welfare has encouraged employers to implement stress management education for all employees to prevent mental disorders and improve work performance [8].

A systematic review of the effects of training programs for work-related stress reported that those using cognitive behavioral therapy (CBT) is the most effective option for alleviating stress [9]. These training programs require time and CBT experts and are conducted not only as therapy but also as stress management training [10-20]. CBT has mainly been implemented as an individual psychotherapy, although low-intensity CBT [21-23] to provide interventions to a relatively larger number of people is becoming increasingly common. Recent programs include the provision of information on CBT via books, group training, and group education. CBT is now also offered via telephone and Web in a self-learning format. This increase in accessibility has proven beneficial in improving stress management [9,24-28]. However, despite these promising findings regarding CBT, its efficacy in the workplace remains uncertain, as the time available for health education of employees in a workplace setting is limited.

Here, we developed a brief educational program based on CBT that is feasible for implementation in a workplace setting. We conducted a pilot study with a randomized controlled design to investigate the effects of the program on alleviation of distress and improvement of stress management abilities in employees who have significant or non-significant distress.

## Methods

### Study Participants and Procedure

The target population consisted of 187 employees (147 men and 40 women) at an information technology company in Tokyo, Japan. The majority of employees were system engineers with a high degree of computer literacy. The company provides in-house training programs for managerial (eg, legal knowledge, human resource management, accounting) and non-managerial positions (eg, health and safety) once or twice a year. The present training program was announced and briefed for the 187 non-management employees in the company. Participation in this study was voluntary, and informed consent was obtained from employees prior to group education after explaining the study purpose, procedures, and details of the training program.

The participants who provided written informed consent were randomly assigned to training or control groups. The training group received group CBT training during working hours in December 2011 and an additional month of CBT homework, with a follow-up study conducted 6 months later. All participants were required to complete self-rated online questionnaires before training, immediately after, and on 6-month follow-up. For ethical reasons, this training program was provided to the control group after follow-up. No exclusion criteria were set, as the study examined the effects of the CBT program in a real-world workplace setting. The study protocol was approved by the Ethics Committee of the School of Allied Health Sciences at Kitasato University. Reporting of methods and results of this study are based on the CONSORT-EHEALTH guidelines [29].

### Contents of the Training Program

The training program was composed of a 150-minute group class presented by a qualified CBT expert on cognitive behavior therapy and 1 month of homework via Web-based CBT program. The following three topics were covered in the group education program: an overview of CBT, problem-solving techniques, and cognitive restructuring techniques. For problem-solving techniques, we encouraged participants to use group brainstorming, which we consider the most important element in a number of problem-solving techniques. For cognitive restructuring techniques, participants received a lecture designed to facilitate understanding of the techniques and column sheets to complete during the Web-based CBT program. Column sheets included the following topics: situations where participants felt stressed, feelings and behaviors, automatic thoughts, an objective examination of those automatic thoughts (including counterevidence), adaptive thoughts, and changes in feeling and behavior. While completing the worksheets, participants held group discussions by exchanging questions and opinions regarding the cognitive restructuring techniques. Participants also improved their understanding of the cognitive restructuring techniques by consulting with the CBT expert. Following group education, the Web-based CBT homework was explained.

**Figure 1:**
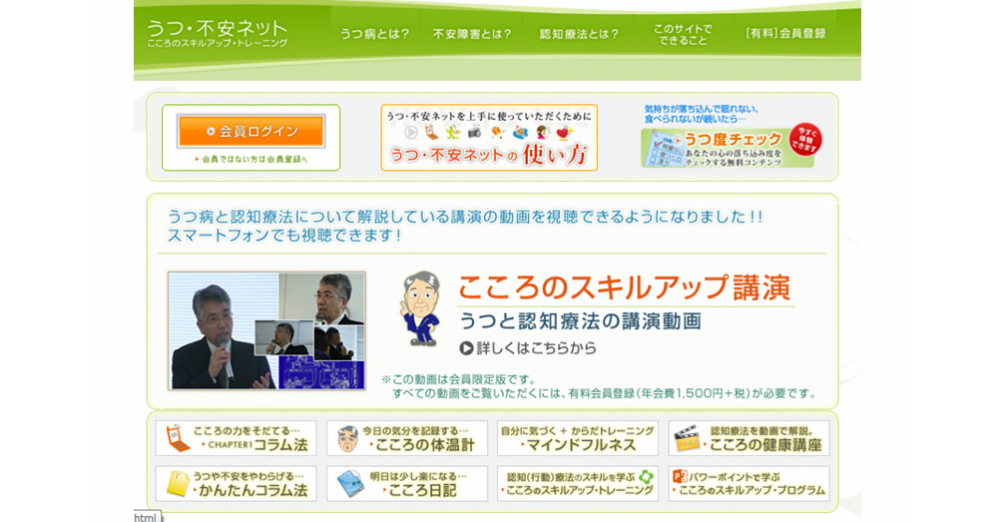
Program screenshot.

**Figure 2:**
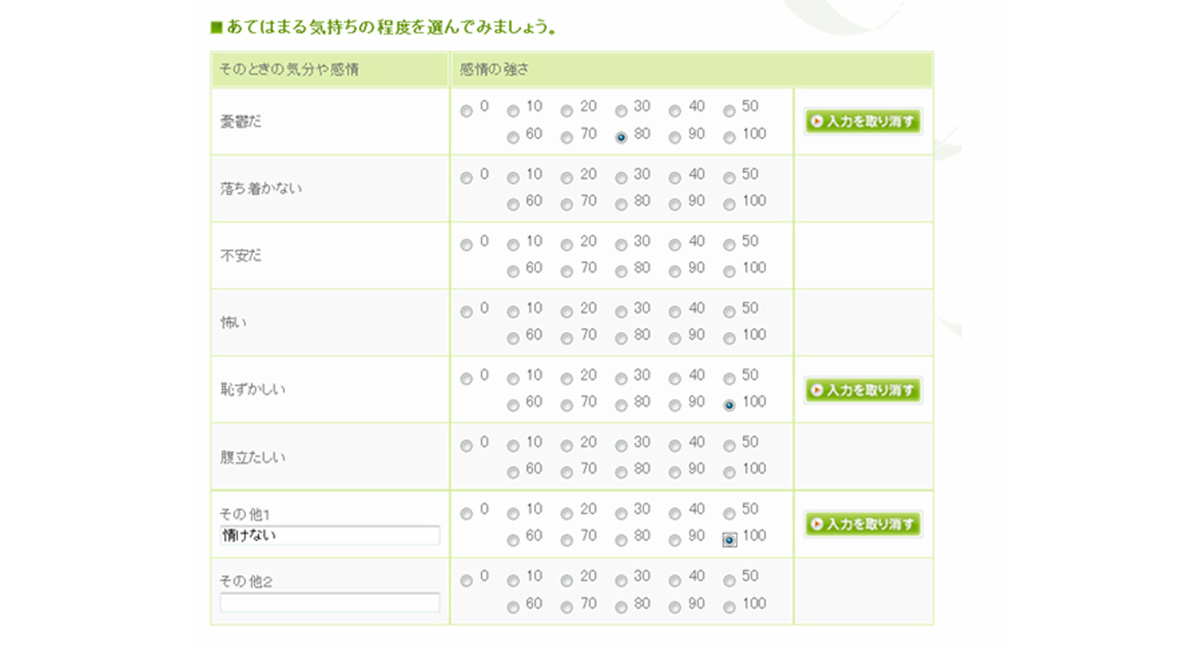
Program screenshot.

Participants were asked to practice the column method with a Web-based CBT program by reflecting on the stress they experienced over a 1-month period starting the day after group education. The Web-based CBT program was developed by Woman Wave Corporation (see Figures 1-2 for screenshots). The fee for the program was prepaid using research expenses to allow participants to access the program free of charge. The recommended Web program enables users to easily complete the column sheets by providing contextual explanations and advice on the column method. Users can complete column sheets by entering information as directed on the screen. In addition to the column sheet above, participants were able to access the program via their computers at work and home and were encouraged to complete their homework at least twice. Homework was expected to help familiarize participants with the column method. However, homework completion was not mandatory, to avoid placing further pressure on participants. To encourage homework completion, occupational health nurses sent a total of four emails, prepared by a CBT expert, to each participant to provide supplementary information and tips regarding the cognitive restructuring techniques. These occupational health nurses received a 150-minute training session before the study from a CBT expert and then answered questions from participants regarding Web-based CBT procedures, with a CBT expert answering any remaining questions. To reduce the burden on participants, the program did not contain homework on problem-solving techniques.

### Outcome Evaluation

The primary outcome was measured as the change in Kessler-6 (K6) score, which measures psychological distress. The K6 score was measured before training to establish a baseline and then immediately after 1 month of training and again after 6 months. Developed as a screening tool for depressive disorders and anxiety disorders [30], the K6 scale is widely used to assess psychological stress [31,32], with the score obtained from a simple self-rating questionnaire on symptoms of depression and anxiety experienced over the previous month. The reliability and validity of the Japanese version of the K6 questionnaire utilized in this study have been verified [33]. A study on the cutoff point to diagnose clinically significant psychological distress of respondents suggest a cutoff point of 4 or 5 (total score, 24) [34].

Secondary outcomes were evaluated based on respondents’ answers to several questions on an original self-rating questionnaire. Questions concerned the recognition of dysfunctional thinking habits (“Do you recognize your dysfunctional thinking habits?”), ability to change dysfunctional thinking patterns to functional ones (“Can you transform your dysfunctional thinking patterns that have been bothering you into functional ones?”), ability to cope with stress (“Are you confident that you can cope with stress by yourself?”), and problem-solving skills (“Do you think you can solve a problem when you face one?”). The respondents answered using a 5-point scale (1=strongly disagree, 5=strongly agree).

### Randomization and Masking

An independent researcher who had no direct contact with the participants used computer-generated randomization with a 1:1 ratio and block size of 6. No stratification was performed and evaluators were masked. Owing to the nature of the intervention, participants were informed of their allocation status.

### Statistical Analysis

A systematic review of the literature on mental disorder intervention suggests that Cohen’s effect size (*d*) for those with sub-threshold depression is 0.42 (95% CI 0.23-0.60) [35]. The sample size necessary to obtain an effect size of 0.42 with probability of Type I error (α) less than .05 and Type II error (β) less than .20 was 90 for each group. A generalized equation was used for estimation, based on an intention-to-treat (ITT) analysis. The rate of missing primary or secondary outcomes was 19.26% across the follow-up period (K6, 18.2%; recognition of dysfunctional thinking habits, 19.6%; changing dysfunctional thinking patterns, 19.3%; coping skills, 19.0%; problem-solving skills, 19.6%). To satisfy the ITT requirement that analyses be conducted for all participants, a multiple imputation (MI) method was used on the assumption that data could be considered missing at random. MI allows for uncertainty caused by missing data by generating several different plausible imputed data sets using a set of external covariates and appropriately combining results obtained from each [36,37]. We utilized a sequential regression approach to generate 20 imputations for each missing value, as recommended by Graham [38].

To determine the effects of the training program, primary and secondary outcomes were measured, and differences in scores before and after implementation for the training and control groups were calculated. The short-term effect was calculated by subtracting the baseline scores from those obtained after completion of 1 month of homework. The long-term effect was calculated by subtracting the baseline scores from those obtained after 6 months. Results are shown as changes in the raw scores for primary and secondary outcomes. In addition, the differences in mean adjusted for baseline score of each outcome were also calculated. For the K6 scale, a sub-analysis was conducted among participants with a K6 score ≥5 at baseline. In addition, the training group was divided into subgroups of those completing Web-based CBT homework at least once and those completing no homework, and changes in the K6 scores of these subgroups were compared.

To analyze baseline characteristics of the study participants, information on sex, age, hours of overtime, mean hours of sleep on weekdays, marital status, drinking habits, exercise habits, and history of psychiatric treatment was collected from each participant at baseline. A *t* test was used for numerical variables and a χ^2^ test for categorical variables. Statistical significance was set at *P*<.05. IBM SPSS Statistics 22 and IBM SPSS Missing Values 22 (*SPSS* Inc., *Chicago*, IL, USA) were used for statistical analyses.

## Results

### Participants

**Figure 3:**
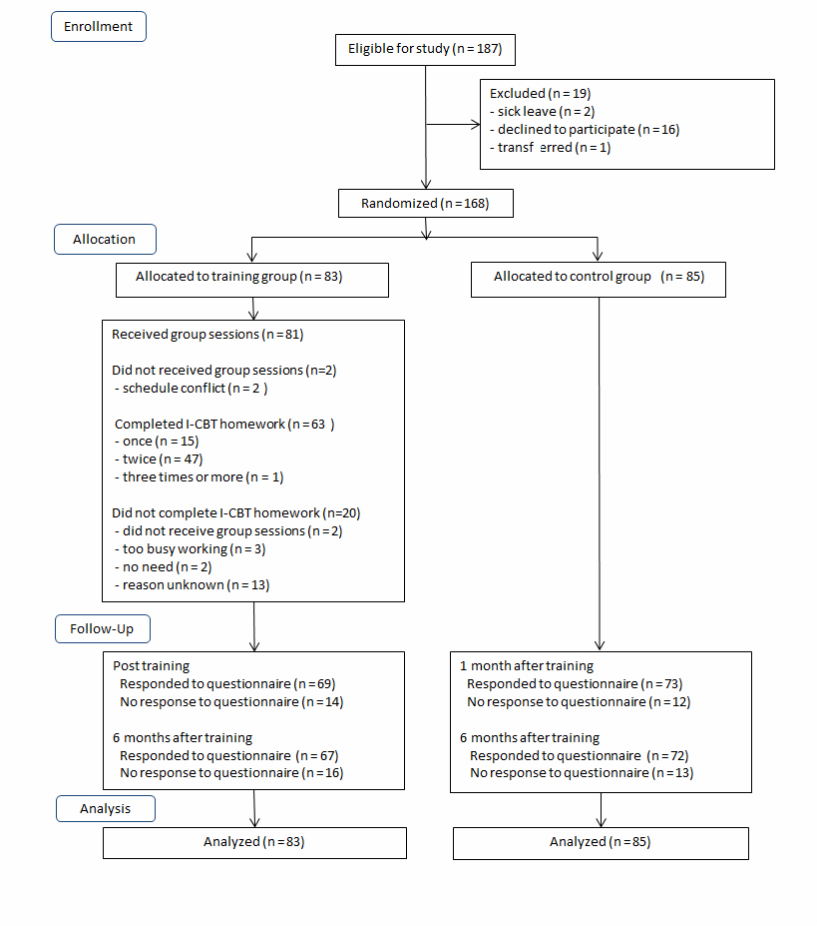
Trial profile.

Figure 3 shows the study flow. Of 187 potential participants, 2 took a leave of absence before the start of the study, 16 did not consent to participate, and 1 was transferred to another location before the study started, leaving 168 enrolled in this study. These 168 participants were randomly assigned to either the training (n=83) or control group (n=85). In the training group, 81 (98%) actually received a group session, and 63 (76%) actually received additional Web-based CBT homework. The completion of homework by participants was as follows: once (n=15), twice (n=47), and more than twice (n=1). Among those who did not complete assigned homework (20/83, 24%), 2 also did not attend the group session.

Follow-up questionnaires immediately after completion of the program were completed by 69 (83%) of the 83 respondents in the training group and by 73 (86%) of the 85 control group participants. Follow-up questionnaires after 6 months were completed by 67 (81%) respondents in the training group and by 72 (85%) in the control group.

Five participants emailed the nurse to inquire about the Web-based CBT. Of these five, two inquired about how to operate the Web program and three about how to complete the column sheets. The content of this advice was the same as that provided in the training session.

### Baseline Characteristics

Baseline characteristics of the 168 participants are shown in Table 1. No missing data were observed. Of the 168 participants, 22% (n=37) were women (19%, 16/83 of training group and 25%, 21/85 of control group), 78% (n=131) were men (81%, 67/83 of training group and 75%, 64/83 of control group), and mean age across both training and control groups was 38.4 years (SD 8.1 and SD 8.4 respectively). No significant differences were observed between the training and control groups regarding sex ratio, mean age, hours of overtime, mean hours of sleep, marital status, exercise and drinking habits, K6 score, ability to recognize and change dysfunctional thinking habits, ability to cope with stress, or problem-solving skills. Although a proportion of participants had pre-existing or ongoing mental health conditions that required psychiatric treatment, this was not significant relative to the populations of the training and control groups. Differences in primary and secondary outcome scores, age, and sex ratio at baseline also did not significantly differ between those who responded to the follow-up questionnaires, both immediately after the study and after 6 months, and those who did not.

**Table 1:** Baseline characteristics of participants in training and control groups.^a,b^

Characteristic		Total (n=168)	Training group (n=83)	Control group (n=85)	*P* value
Men, n (%)		131 (78.0)	67 (80.7)	64 (75.3)	.26
Age, years, mean (SD)		38.4 (8.3)	38.4 (8.1)	38.4 (8.4)	.35
**Overtime hours, n (%)**					
	Few	54 (32.1)	28 (33.7)	26 (30.6)	.34
	<40 hours	97 (57.7)	46 (55.4)	51 (60.0)	
	40-79 hours	17 (10.1)	9 (10.8)	8 (9.4)	
	≥ 80 hours	0 (0)	0 (0)	0 (0)	
**Mean hours of sleep (weekday), n (%)**					
	<5 hours	17 (10.1)	9 (10.8)	8 (9.4)	.84
	5-6 hours	124 (73.8)	59 (71.1)	65 (76.5)	
	7-8 hours	26 (15.5)	15 (18.1)	11 (13.1)	
	≥9 hours	1 (0.60)	0 (0)	1 (1.20)	
**Marital status, n (%)**					
	Married	77 (45.8)	41 (49.4)	36 (42.4)	.41
	Single	91 (54.2)	42 (50.6)	49 (57.6)	
**Drinking, n (%)**					
	None	64 (38.1)	33 (39.8)	31 (36.5)	.29
	1-3 days/week	61 (36.3)	26 (19.3)	35 (41.2)	
	4-6 days/week	19 (11.3)	10 (12.0)	9 (10.6)	
	Every day	24 (14.3)	14 (16.9)	10 (11.8)	
**Exercise habit, n (%)**					
	None	88 (52.4)	41 (49.4)	47 (55.3)	.6
	1-2 times/week	65 (38.7)	35 (42.2)	30 (35.3)	
	≥3 times/week	15 (8.90)	7 (8.40)	8 (9.40)	
**History of psychiatric treatment, n (%)**					
	No history	143 (85.1)	69 (83.1)	74 (87.1)	.28
	History of treatment	16 (9.50)	8 (9.60)	8 (9.40)	
	Undergoing treatment at present	9 (5.40)	6 (7.20)	3 (3.50)	
K6 score, mean (SD)		4.8 (4.50)	4.7 (4.50)	4.8 (4.50)	.90
Recognition of dysfunctional thinking habits, mean (SD)		2.36 (0.95)	2.53 (1.02)	2.20 (0.87)	.57
Changing dysfunctional thinking patterns, mean (SD)		3.17 (0.89)	3.10 (0.91)	3.24 (0.86)	.33
Coping skills, mean (SD)		3.08 (0.93)	3.10 (0.90)	3.06 (0.96)	.49
Problem-solving skills, mean (SD)		2.87 (0.94)	2.82 (0.97)	2.92 (0.91)	.54

^a^Independent *t* test for difference between groups for continuous measures and chi-square test for differences between groups for categorical characteristics.

^b^Scores on a scale 1-5, with 1 indicating not at all and 5 indicating very well.

### Effects of Training Program

Table 2 shows the results of ITT analysis. From before to immediately after training, the mean K6 score of the training group decreased by 0.46 while that of the control group increased by 0.22. However, this difference was not significant (mean difference 0.68, 95% CI −0.44 to 1.80). The intergroup difference in change in mean K6 score for the training and control groups from baseline to 6 months after program completion was also not significant (−0.14 at baseline, 0.83 after 6 months; mean difference 0.97, 95% CI −0.64 to 2.59). No significant effect due to the training program was observed, even when K6 scores were adjusted for baseline scores. Further, while ability of participants to recognize their own dysfunctional thinking was significantly increased both immediately after training (mean difference 0.33, 95% CI 0.06-0.59; *d*=0.37) and at 6 months (mean difference 0.45, 95% CI 0.06-0.83; *d*=0.33), significance was not observed after adjusting for baseline scores of recognition of dysfunctional thinking habits. The ability of participants to transform thought patterns from dysfunctional to functional was also not improved immediately after training in the training group. However, after adjustment for baseline scores, a small significant difference was observed immediately after training, but not after 6 months. While the ability of participants to cope with stress was not significantly improved immediately after training, significant improvement was noted after 6 months in the training group (mean difference 0.54, 95% CI 0.10-0.98; *d*=0.37), a finding that remained even after adjustment for baseline scores. The problem-solving ability of participants was not improved either immediately after training or after 6 months.

At baseline, 36 participants in the training group (43%, 36/83) and 37 in the control group (44%, 37/85) exhibited clinically significant psychological distress (K6 score ≥5). Results of subgroup analysis are shown in Table 3. Mean K6 score was significantly improved in the training group compared with the control group immediately after training (−2.50 vs −0.07; mean difference 2.43, 95% CI 0.55-4.31; *d*=0.61), and this effect remained even after 6 months (−3.49 vs −0.50; mean difference 2.99, 95% CI 0.70-5.29; *d*=0.60) and on adjustment for baseline scores. In addition, the 36 participants of the training group with K6 cutoff score ≥5 points were subdivided into a complete group (n=28) who completed at least 1 homework session and an incomplete group (n=8) who completed no sessions. In contrast to the 37 participants in the control group with a baseline K6 score ≥5, the Web-based CBT complete subgroup had significantly lower mean scores both immediately after training (−2.60 vs −0.07; mean difference 2.53, 95% CI 0.46-4.60; *d*=0.63) and 6 months later (−4.02 vs −0.50; mean difference 3.52, 95% CI 1.13-5.90; *d*=0.74). The same result was observed when scores were adjusted for baseline scores. In addition, mean K6 score for those completing no homework (or Web-based CBT incomplete subgroup) was lower, though not significantly, immediately after training (−2.14 vs −0.07; mean difference 2.07, 95% CI −0.65 to 4.80; *d*=0.53) but increased 6 months later with no difference being observed from the control group (−1.67 vs −0.50; mean difference 1.16, 95% CI −3.44 to 5.77; *d*=0.23). The same result was observed when scores were adjusted for baseline scores.

### Study Safety

This study did not exacerbate any existing psychological problems of any participants. While one subject did experience mild distress during group education after being reminded of painful memories, they recovered quickly.

**Table 2:** Intention-to-treat analyses of primary and secondary outcomes after training and 6-month follow up.

Variable		Immediately after training				6-month follow-up			
		Mean (SE) change	Unadjusted difference in mean (95% CI)	Adjusted difference in mean (95% CI)^a^	Effect size^b^	Mean (SD) change	Unadjusted difference in mean (95% CI)	Adjusted difference in mean (95% CI)^a)^	Effect size^b^
**K6 scores**									
	Training group	−0.46 (0.44)	0.68 (−0.44 to 1.80)	0.70 (−0.34 to 1.73)	0.19	−0.14 (0.64)	0.97 (−0.64 to 2.59)	0.99 (−0.45 to 2.44)	0.18
	Control group	0.22 (0.34)				0.83 (0.55)			
**Recognizing dysfunctional thinking habits**									
	Training group	0.40 (0.10)	0.33 (0.06-0.59)^c^	0.19 (−0.02 to 0.40)	0.37	0.68 (0.15)	0.45 (0.06-0.83)^c^	0.26 (−0.04 to 0.56)	0.33
	Control group	0.08 (0.09)				0.24 (0.14)			
**Changing dysfunctional thinking patterns**									
	Training group	0.27 (0.08)	0.18 (−0.04 to 0.40)	0.23 (0.02-0.44)^c^	0.26	0.43 (0.20)	0.23 (−0.28 to 0.74)	0.31 (−0.12 to 0.75)	0.13
	Control group	0.08 (0.08)				0.21 (0.17)			
**Coping skills**									
	Training group	0.21 (0.10)	0.23 (−0.02 to 0.48)	0.21 (−0.02 to 0.45)	0.27	0.77 (0.16)	0.54 (0.10-0.98)^c^	0.53 (0.15-0.91)^d^	0.37
	Control group	−0.01 (0.08)				0.23 (0.16)			
**Problem-solving skills**									
	Training group	0.12 (0.12)	0.17 (−0.13 to 0.47)	0.17 (−0.12 to 0.45)	0.18	−0.01 (0.20)	0.11 (−0.43 to 0.64)	0.06 (−0.41 to 0.54)	0.06
	Control group	−0.06 (0.10)				−0.12 (0.19)			

^a^Adjusted for baseline scores.

^b^Unadjusted Cohen’s *d*.

^c^ *P*<.05.

^d^ *P*<.01.

**Table 3:** Subanalyses of K6 score among participants with K6 ≥5 at baseline.

Subgroup	Immediately after training				6-month follow-up			
	Mean (SE) change	Unadjusted difference in mean (95% CI)	Adjusted difference in mean (95% CI)^a^	Effect size^b^	Mean (SD) change	Unadjusted difference in mean (95% CI)	Adjusted difference in mean (95% CI)^a^	Effect size^b^
Training group (all), n=36	−2.50 (0.67)	2.43 (0.55-4.31)^c^	2.45 (0.70-4.20)^d^	0.61	−3.49 (0.89)	2.99 (0.70-5.29)^c^	3.02 (0.87-5.17)^d^	0.60
Training group (completed homework^e^), n=28	−2.60 (0.78)	2.53 (0.46-4.60)^c^	2.60 (0.67-4.53)^d^	0.63	−4.02 (0.93)	3.52 (1.13-5.90)^d^	3.60 (1.42-5.78)^d^	0.74
Training group (did not complete homework), n=8	−2.14 (1.25)	2.07 (−0.65 to 4.80)	1.93 (−0.58 to 4.45)	0.53	−1.67 (2.25)	1.16 (−3.44 to 5.77)	1.00 (−3.27 to 5.28)	0.23
Control group, n=37	−0.07 (0.65)	-	-	-	−0.50 (0.77)	-	-	-

^a^Adjusted for sex, age, and baseline K6 scores.

^b^Unadjusted Cohen’s *d* compared to control group.

^c^ *P*<.05.

^d^ *P*<.01.

^e^Did homework using I-CBT more than once (mean implementation times was 1.5).

## Discussion

### Principal Findings

The results of this study suggest that a brief stress management program combining group CBT training and Web-based CBT homework does not provide significant alleviation of stress when analyzed across all participants but does provide moderate alleviation of symptoms in employees with clinically significant psychological distress. Our results further suggest that this type of educational program can improve self-confidence in the ability to cope with stress.

In previous studies of CBT for psychiatric patients, considerable time was required for face-to-face interaction between the CBT expert and patients, and intervention was discontinued in 30% to 50% of cases [24,39,40]. This conventional method is therefore generally considered unfeasible in a workplace setting, as most employees have insufficient time to complete high-intensity CBT and are considered less motivated regarding participation in CBT than psychiatric patients in a clinical setting.

Recently, low-intensity CBT programs have become increasingly popular, and such programs have been implemented to provide medical and psychological support to as many people experiencing psychological distress as possible [21-23]. The positive effects of low-intensity CBT on the mental health of patients have already been reported for group education [41] as well as for therapy via email, phone [24,25], and Internet [26-28]. Further, the discontinuation rate for low-intensity CBT is lower than that for face-to-face CBT [24,42-44].

Van der Klink et al reviewed 49 studies to compare the effects of cognitive-behavioral, multimodal, relaxation, and organization-focused intervention programs [9] on work-related stress. Improvement in psychological distress was greatest for cognitive-behavioral intervention programs such as CBT (*d*=0.68), followed by multimodal programs (*d*=0.51), relaxation programs (*d*=0.35), and organization-focused programs (*d*=0.08). While the intensity of CBT reviewed by Van der Klink et al was lower than conventional CBT, it is probably still unsuitable for many workplaces as a high degree of care, time, and involvement by experts is required. Of note, in Van der Klink et al’s review, the mean number of CBT sessions was 7.6 [9]. Mohr et al reported that face-to-face CBT sessions and CBT sessions via telephone were conducted a total of 16 times, with a mean session time of approximately 45 minutes in their study [24]. The protocol for Web-based CBT by Titov et al required six sessions across 8 weeks, and experts held these sessions on a one-on-one basis [45]. Similarly, in a study of T-CBT by Furukawa et al, 30- to 45-minute sessions were held a total of eight times, and experts (such as therapists) were available for each participant, although no one-on-one sessions were required [46].

The training program implemented in the present study was developed by combining a group CBT course with short-term Web-based CBT. To enable implementation in a workplace setting, the program was simplified even more than conventional low-intensity CBT. Concern of benefits being compromised due to oversimplification is therefore justified. Indeed, although a brief Web-based psychoeducational program was found to have slightly improved the degree of occupational satisfaction, it did not clearly improve self-efficacy or problem-solving skills [47]. To counteract any detractions due to the simplicity of our program, we increased efficacy by asking participants to complete individual homework assignments.

In the present study, analysis across all participants demonstrated alleviation of stress without statistical significance. Two possible reasons for this lack of a substantial finding are that some participants may not have clearly understood the requirements for training, subsequently losing motivation to participate in the CBT program. However, despite the lack of any notable effect of our educational program across all participants, we found that our program did significantly alleviate the stress among those employees who had clinically significant psychological distress before the program. This positive effect was particularly high in the training subgroup that also completed Web-based CBT homework, likely due to the stronger motivation of employees with higher stress to participate and actively complete the homework, thereby alleviating stress. Participants who were provided information concerning CBT during the Web-based CBT are reportedly more likely to complete their homework [48,49]. During the present study, participants were provided CBT-related information four times during the Web program, which might have enhanced their understanding of CBT and thereby increased the positive effects of CBT among those who completed the Web-based CBT homework.

Analysis across all participants shows that the ability of participants to recognize dysfunctional thinking habits increased significantly both immediately after training and on 6-month follow-up. However, these differences disappeared after adjusting for baseline data. The ability to correct dysfunctional thinking patterns between two groups was only significantly different on 6-month follow-up after adjustment. The poor robustness of these results might be due to the lack of statistical power. We are therefore unable to conclude whether or not the ability to recognize dysfunctional thinking habits and correct dysfunctional thinking patterns improved in the present study. Nevertheless, the ability to cope with stress significantly improved on 6-month follow-up in both unadjusted and adjusted analyses. These results suggest that this program improves confidence in stress management skills. Using CBT to teach this new stress management skill to participants might improve various health outcomes [50-52].

Regarding problem-solving skills, participants might have been unable to develop these skills due to the relatively short period of time allocated during group education and lack of Web-based CBT homework on the subject. Lecture sessions (with group work) and homework assignments therefore appear necessary to help participants acquire these problem-solving skills.

### Limitations

Several limitations to the present study may prevent our findings from being fully generalizable. First, participants were employees of an information technology company and might therefore have been more likely to consent to Web-based CBT than the general population. Second, the reliability and validity of the original question items have not been evaluated. Therefore, the results of secondary outcomes cannot be confirmed. Third, this study was non-blinded and the participants in the training and control groups worked in the same office and might have shared information. Fourth, we evaluated one primary outcome and four secondary outcomes, which might have increased the possibility of a Type I error. Finally, the sample size was insufficient because we calculated sample size necessary to obtain an effect size of 0.42. Further, the target population contained a significant number of distressed subjects and participation rate was lower than originally expected, which could have affected the robustness of statistical results.

Validation of the effects of this brief training program on the alleviation of distress and development of stress management in the workplace will require randomized clinical trials in a variety of workplaces with diverse corporate structures.

### Conclusions

The results of this study suggest that a brief stress management program combining group CBT and a Web-based CBT can have positive effects on the alleviation of symptoms in employees with clinically significant psychological distress and development of confidence to cope with stress. These brief educational programs based on CBT principles might be an effective preventive measure for addressing current concerns of presenteeism and absenteeism among employees with high levels of stress.

## Abbreviations

CBT: cognitive behavioral therapy
ITT: intention-to-treat
K6: Kessler-6 scale
MI: multiple imputation
